# The Absorption, Distribution and Excretion of ^3^H-Chlorambucil in Rats Bearing the Yoshida Ascites Sarcoma

**DOI:** 10.1038/bjc.1971.96

**Published:** 1971-12

**Authors:** Bridget T. Hill, Pamela G. Riches

## Abstract

The distribution of ^3^H-chlorambucil following its administration by subcutaneous injection to Yoshida ascites tumour-bearing rats has been examined, in an attempt to elucidate the metabolic fate and mode of action of this drug.

Drug uptake into the body tissue was rapid, with a high level of radioactivity being associated with the plasma and ascitic fluid during the initial 6-hour period after treatment. Previous studies *in vitro* had shown that chlorambucil-resistant cells accumulated less drug than their sensitive counterparts: this discrepancy was also observed after *in vivo* drug treatment and was reflected in the two-fold difference in extent of binding of tritium to DNA, RNA and protein isolated from the 2 cell strains. These results might in part explain the observed difference in metabolism of chlorambucil by the resistant and sensitive strain of Yoshida ascites sarcoma cells.


### THE ABSORPTION, DISTRIBUTION AND EXCRETION OF $^{3}H$-CHLORAMBUCIL IN RATS BEARING THE YOSHIDA ASCITES SARCOMA

BRIDGET T. HILL AND PAMELA G. RICHES

*From the Department of Applied Biochemistry, Chester Beatty Research Institute, Institute of Cancer Research: Royal Cancer Hospital, Fulham Road, London SW3 6JB*

Received for publication September 29, 1971

**SUMMARY.—The distribution of $^{3}H$-chlorambucil following its administration by subcutaneous injection to Yoshida ascites tumour-bearing rats has been examined, in an attempt to elucidate the metabolic fate and mode of action of this drug.**

**Drug uptake into the body tissue was rapid, with a high level of radioactivity being associated with the plasma and ascitic fluid during the initial 6-hour period after treatment. Previous studies *in vitro* had shown that chlorambucil-resistant cells accumulated less drug than their sensitive counterparts: this discrepancy was also observed after *in vivo* drug treatment and was reflected in the two-fold difference in extent of binding of tritium to DNA, RNA and protein isolated from the 2 cell strains. These results might in part explain the observed difference in metabolism of chlorambucil by the resistant and sensitive strain of Yoshida ascites sarcoma cells.**

CHLORAMBUCIL has been used extensively for the treatment of chronic lymphoblastic leukaemia (Boesen *et al.*, 1964), but the development of resistance to the drug has restricted its usefulness. An understanding of the factors responsible, particularly its mode of action, transport and tissue distribution might lead to a wider application of this drug in cancer chemotherapy. At present there is no detailed information concerning these factors, in either experimental animals or men.

An *in vivo* study of the tissue and cellular distribution of isotopically-labelled chlorambucil (586 mCi/mmole) in rats bearing a chlorambucil-sensitive or chlorambucil-resistant Yoshida ascites sarcoma has therefore been made in an attempt to elucidate the metabolic fate and mode of action of this drug.

### MATERIALS AND METHODS

Full details of tumour transplantation techniques have been described previously (Harrap and Hill, 1969). Tritium-labelled chlorambucil 4-(4-di-(2-chloroethyl)amino-3,5-$^{3}H$ phenyl)butyric acid was synthesized by the reductive tritiation of the iodinated derivative in the Chester Beatty Research Institute (Jarman, unpublished work, 1970). The drug was administered subcutaneously to animals bearing the Yoshida ascites sarcoma in ascitic form at a dose of 8 mg./kg. body weight on the fourth day following tumour transplantation because this dose induced complete regression of the sensitive tumour but was without effect on the growth rate of the resistant tumour (Harrap and Hill, 1969).

Reagent chemicals were obtained from Hopkin and Williams Ltd. or British Drug Houses Ltd., AnalaR grades being used where available.

*Metabolic studies*

At intervals after drug administration, animals were anaesthetized with ether, and blood samples removed by cardiac puncture using heparin as the anticoagulant (10 i.u./ml.). Plasma samples were obtained by centrifugation of whole blood at 350 ***g*** for 15 minutes at 4° C. The animals were killed and ascitic fluid collected and separated from tumour cells by centrifugation. The tumour cells remaining in the peritoneum were aspirated with ice-cold 0·3% saline, washed in a solution of phosphate-buffered saline (PBS)* and resuspended to a known volume. The cell concentration was determined using a modified Fuchs–Rosenthal counting chamber. Tissue samples were removed from kidney, heart, liver, spleen, lung and gastrocnemius muscle, and their wet weights determined by difference using pre-weighed sample tubes.

The excretory rates of tritium-labelled material under these experimental conditions were determined by collecting urine and faeces separately at various time intervals from rats confined in glass metabolism cages (Jencons).

*Radioactive counting*

Plasma and ascitic fluid were diluted 3-fold, and whole blood 30-fold, and solubilized in 12·5% aqueous tetraethylammonium hydroxide (TEH). Solid tissues (approximately 50 mg. wet weight) were oxidized to completion using a Packard TriCarb sample Oxidizer. Aliquots of cell suspensions containing approximately $5 \times 10^7$ cells were centrifuged at 500 ***g*** (4°) and washed twice with 5 ml. volumes of ice-cold PBS. The resultant cell pellets were then either

(a) Dissolved in 12·5% aqueous TEH (for measurement of gross drug uptake by the cells), or

(b) Extracted by shaking for 1 minute successively with $2 \times 2$ ml. volumes of ethanol (4°), allowed to stand at 0° for 30 minutes before removing and keeping the ethanol supernatant fraction, or

(c) Subjected to a modification of the Schmidt–Thannhauser technique to obtain DNA, RNA and protein containing fractions (Munro and Fleck, 1966).

Aliquots of these extracts were assayed for radioactivity using a Toluene–Phosphor scintillant in a Packard TriCarb Liquid Scintillation Counter Model 3375.

DNA was estimated according to Burton (1956), RNA by the orcinol procedure (Brown, 1946) and protein by the method of Lowry, Rosebrough, Farr and Randall (1951).

Each calculated figure represents the mean value of duplicate experimental samples from 3 animals.

### RESULTS

Following subcutaneous injection of $^3$H-chlorambucil, drug uptake into the body tissues was rapid. After 1 hour's exposure to the drug, of the tissues studied, the highest $^3$H-concentration was found in the liver. The tissue concentrations

* The following abbreviations will be used throughout this paper: PBS—phosphate buffered saline: TEH—tetraethylammonium hydroxide.

of labelled material expressed as a fraction of the corresponding liver value ($86 \times 10^3$ DPM/mg. wet weight of tissue) are shown in Table I. It should be noted that after this initial interval (1 hour after treatment) the concentration of tritium increased in kidney, but the levels in the other tissues decreased with respect to time, the tritium being cleared most rapidly from the liver.

In whole blood the radioactive label was mainly associated with the plasma: the tritium concentration in blood and ascitic fluid was similar. A high level of radioactivity was associated with these fluids throughout the first 6-hour period following treatment (Table II).

In none of these measurements were there significant differences in the extent of drug binding to tissues or fluids in animals bearing either the drug-sensitive

TABLE I.—*Distribution of Radioactivity in Yoshida Ascites Tumour Bearing Rats after a Single Subcutaneous Injection of 8 mg./kg. $^3$H-Chlorambucil Expressed as a Fraction of the (Initial) Liver Values at 1 hour in DPM/mg. wet weight*

| | Time after drug administration (hours) | | |
|---|---|---|---|
| Organ | 1 | 6 | 48 |
| Liver* | 1·00 | 0·48 | 0·17 |
| Lung | 0·22 | 0·14 | 0·10 |
| Kidney | 0·63 | 0·88 | 0·51 |
| Heart | 0·11 | 0·10 | 0·07 |
| Muscle | 0·07 | 0·06 | 0·04 |
| Spleen | 0·06 | 0·07 | 0·06 |
| Resistant ascites tumour cells | 0·04 | 0·05 | 0·07 |

* Liver contained $86 \cdot 2 \times 10^3$ DPM/mg. wet weight 1 hour after drug treatment. Assuming all the tritium were associated with chlorambucil, this value would be equivalent to $7 \times 10^{-9}$ mmoles chlorambucil per mg. wet weight of tissue.

TABLE II.—*Distribution of Radioactivity in Plasma and Ascitic Fluid from Rats Bearing the Yoshida Ascites Sarcoma Following a Single Subcutaneous Injection of $^3$H-Chlorambucil*

| DPM/ml. fluid | Time after drug administration (hours) | | | | |
|---|---|---|---|---|---|
| $\times 10^6$ | 1 | 6 | 12 | 24 | 48 |
| Ascitic fluid | 6·6 | 8·1 | 2·6 | 1·9 | 1·8 |
| Plasma | 4·3 | 6.7 | 2·5 | 2·2 | 2·1 |

or -resistant tumours. However, differences were apparent when the total cellular uptake of the drug and its binding to nucleic acids and protein in the tumour cells was determined. The concentration of tritium in the sensitive and resistant cells was similar during the first hour after drug administration (Fig. 1). The level in the resistant cells increased slightly (20%) to a maximum value approximately 6 hours after treatment, whilst the level associated with the sensitive cells doubled during this time interval. After this period the $^3$H-concentration in both cell strains decreased, but the sensitive cells still maintained 2–3 times more tritium than the resistant cells.

It has been shown that free chlorambucil (*i.e.* not protein or nucleic acid bound) can be extracted from tumour cells by treatment with ethanol (Hill, Jarman and Harrap, 1971); 80% of the total tritium counts associated with the cells were extractable by this procedure 1 hour after subcutaneous drug injection. This percentage decreased with time (Table III). But even at the period of maximum uptake of labelled material by the cells (6 hours after treatment) 50% of the counts could be extracted into ethanol.

Peak binding of $^3$H-chlorambucil to proteins and nucleic acids of ascites cells occurred at discrete time intervals in the 2 tumour strains, namely 6 and 12 hours after treatment in the drug-resistant and drug-sensitive cells respectively. This peak binding of tritium to sensitive cells occurred later than the period of maximal drug uptake by whole cells. Both cell strains had similar quantities of tritium associated with their nucleic acids and proteins during the first hour after drug

FIG. 1.—Radioactivity associated with the ascites tumour cells following a single subcutaneous injection of $^3$H-chlorambucil (8 mg./kg.) to tumour-bearing rats. ●——● sensitive cells, ○——○ resistant cells. Each point represents the mean from 3 separate animals—overall scatter at each point, 10%.

injection, but after this period the sensitive cells were shown to contain approximately 2–3 times more bound drug than the resistant cells. In sensitive and

TABLE III.—*The Percentages of the Total Tritium Counts Associated with the Yoshida Ascites Tumour Cells which were Extractable into Ethanol, after a Single Subcutaneous Injection of $^3$H-Chlorambucil into Tumour-bearing Animals*

| Time after injection (hours) | % of total $^3$H-counts per cell extractable into ethanol | |
|---|---|---|
| | Sensitive tumour cells | Resistant tumour cells |
| 1·0 | 82 | 84 |
| 6·0 | 47 | 52 |
| 12·0 | 28 | 27 |
| 24·0 | 16 | 18 |

resistant cells the RNA appeared to be more extensively labelled than the DNA and protein (Fig. 2).

The $^3$H activity recovered in the urine following a single dose of $^3$H-chlorambucil amounted to 33% of the total administered dose when the urine had been collected for a period of 6 hours and to about 60% after 24 hours. Negligible quantities of the tritium in urine were shown to be associated with tritiated water, since a similar recovery of tritium was obtained after

(i) direct measurement of tritium in urine, and
(ii) re-estimation of its tritium content after reducing urine to dryness under vacuum and reconstituting with water.

Tritium associated with the faecal pellets over the 24 hour period was less than 0·2% of the total administered dose. No differences in excretory patterns of $^3$H-labelled materials were observed between animals bearing the 2 tumour strains of tumour.

FIG. 2.—Distribution of radioactivity between DNA, RNA and protein (isolated by a modified Schmidt-Thannhauser procedure) from drug-sensitive and -resistant Yoshida ascites cells following a single subcutaneous injection of $^3$H-chlorambucil (8 mg./kg.) to tumour-bearing rats. ●——● sensitive cells, ○——○resistant cells. Each point represents the mean from 3 separate animals—overall scatter at each point, 10%.

### DISCUSSION

The data presented above are concerned only with measurements of tritium concentrations in the body tissues and no attempt has been made in these experiments to identify any of the chlorambucil metabolites with which the tritium label may be associated. A detailed study of these metabolites is at present under investigation in these laboratories.

The results of the study described here indicate a relatively uniform distribution of tritium after the administration of $^3$H-chlorambucil to tumour-bearing rats, in accord with the data of Milner, Klatt, Young and Stehlin (1965), who failed to detect any significant localization of isotopes in specific tissues in studies with 2-chloroethyl derivatives. In these experiments tritium concentrated primarily in liver and kidney. This was anticipated since a function of these organs is to protect the host from any adverse effects of foreign substances. Accumulation of label in most organs was maximal within 1 hour except in kidney where the concentration of tritium increased during the initial 24-hour period of study.

The progressive rapid loss of label from the liver after initial uptake, was similar to data presented by Connors and Melzack (1971) using CB 1954 and Ball, Connors, Double, Ujhazy and Whisson (1966) using Melphalan. While approximately 60% of the administered radioactivity had been excreted in the urine 24 hours after treatment, of the remaining 40%, approximately 33% could be accounted for in terms of the tritium associated with the body tissues: there was negligible excretion *via* the faeces. Measurements of exhaled tritium were not made, neither was an estimate of the tritium concentration in the brain or cerebro-spinal fluid obtained.

More drug entered the sensitive tumour cells than the resistant cells, which confirms previous data obtained using *in vitro* systems (Harrap and Hill, 1970; Hill, Jarman and Harrap, 1971). However, after the *in vivo* treatment described above, the initial uptake of the drug by the cells was similar (*i.e.* 1 hour after treatment). Only after this period of time was the difference between the 2 cell strains apparent. These results might be explained by reference to the different metabolism of the drug by these cells *in vitro*, namely that the resistant cells have a greater ability to hydrolyse and metabolize the drug (Harrap and Hill, 1970).

Maximal uptake of labelled compound by the cells did not occur until 6 hours after treatment: this was also the time of peak concentration of label in plasma and ascitic fluid. During this initial period the greater proportion of $^3H$-labelled material associated with the cells was shown to be extractable into ethanol: this was in agreement with previous *in vitro* studies in which a " lipophylic-association " of chlorambucil was demonstrated (Hill, Jarman and Harrap, 1971). Furthermore, it is only after, or coincident with this period that peak binding to cell macromolecules in both cell strains is noted, which would support the hypothesis that there can be slow release from this lipophylic association followed by subsequent binding to protein and nucleic acids. These data would suggest that chlorambucil may exert a cytotoxic effect by intracellular binding over a much longer period than might be assumed from its short chemical half-life and rapid rate of hydrolysis (Ross, 1962), especially in the drug-sensitive ascites cell. These findings may also be related to the clinical observation that chemotherapy with chlorambucil only produces a maximal effect several months after its administration to the patient (Boesen and Davis, 1969). Attempts are now being made to isolate the metabolites produced and to characterize more fully the identity of the compound which possesses these lipophylic properties (*i.e.* whether under these conditions it retains an active mustard group). The fact that peak cellular binding of tritium in sensitive cells occurred only 12 hours after treatment, which is much later than with Melphalan (Ball *et al.*, 1966), may also serve to emphasize individual differences between alkylating agents and could be related to the much delayed effects that chlorambucil, in contrast to Melphalan, has on the growth rates of the chlorambucil-sensitive Yoshida ascites cells (Harrap and Hill, 1969): in drug-sensitive tumour-bearing animals, no effect on the growth is observed until 24 hours after treatment.

Whilst peak macromolecular binding in the sensitive cells occurred only 6 hours after maximum drug uptake, this delay was not apparent in the resistant cells, with peak binding occurring 6 hours after treatment; this effect may be associated with differing metabolism of the drug, and more details of the identity of the binding species must be obtained in order to determine the significance of these findings.

Work with 1-(2-chloroethyl)-3-cyclohexyl-1-nitrosourea (Oliverio, Vietzke, Williams and Adamson, 1970) has demonstrated that it is the cyclohexyl portion of the molecule and not the chloroethyl segment which is bound to protein. Since the active mustard group of chlorambucil cannot be detected in the plasma or urine 6 hours after treatment, this may mean that the binding after treatment which occurs at this period, and subsequently in the cells, may also involve another part of the chlorambucil molecule other than the 2-chloroethyl groups.

The authors wish to thank Dr. K. R. Harrap for much helpful advice and discussion, Professor A. B. Foster and Dr. M. Jarman for the supply of labelled chlorambucil, and Mrs. S. Bower and Mrs. M. Clarke for technical assistance.

This investigation has been supported by grants to the Chester Beatty Research Institute (Institute of Cancer Research: Royal Cancer Hospital) from the Medical Research Council and the Cancer Research Campaign. One of us (B.T.H.) acknowledges the receipt of a Wellcome Foundation postdoctoral fellowship.

### REFERENCES

BALL, C. R., CONNORS, T. A., DOUBLE, J. A., UJHAZY, V. AND WHISSON, M. E.—(1966) *Int. J. Cancer*, **1**, 319.

BOESON, E. AND DAVIS, W.—(1969) In ' Cytotoxic Drugs in the Treatment of Cancer '. London (Arnold), p. 89.

BOESEN, E., GALTON, D. A. G. AND WILTSHAW, E.—(1964) In ' Chemotherapy of Cancer ', edited by Plattner. London (Elsevier), pp. 51–61.

BROWN, A. M.—(1946) *Archs Biochem.*, **11**, 269.

BURTON, K.—(1956) *Biochem. J.*, **62**, 315.

CONNORS, T. A. AND MELZACK, D. H.—(1971) *Int. J. Cancer*, **7**, 86.

HARRAP, K. R. AND HILL, B. T.—(1969) *Br. J. Cancer*, **23**, 210, 227.—(1970) *Biochem. Pharmac.*, **19**, 209.

HILL, B. T., JARMAN, M. AND HARRAP, K. R.—(1971) *J. med. Chem.*, **14**, 614.

LOWRY, O., ROSEBROUGH, N. J., FARR, A. L. AND RANDALL, J.—(1951) *J. biol. Chem.*, **193**, 265.

MILNER, A. N., KLATT, O., YOUNG, S. E. AND STEHLIN, J. S. JR.—(1965) *Cancer Res.*, **25**, 259.

MUNRO, H. N. AND FLECK, A.—(1966) *Meth. biochem. Analysis*, **14**, 133.

OLIVERIO, V. T., VIETZKE, W. M., WILLIAMS, M. K. AND ADAMSON, R. H.—(1970) *Cancer Res.*, **30**, 1330.

ROSS, W. C. J.—(1962) In ' Biological Alkylating Agents '. London (Butterworth and Company).


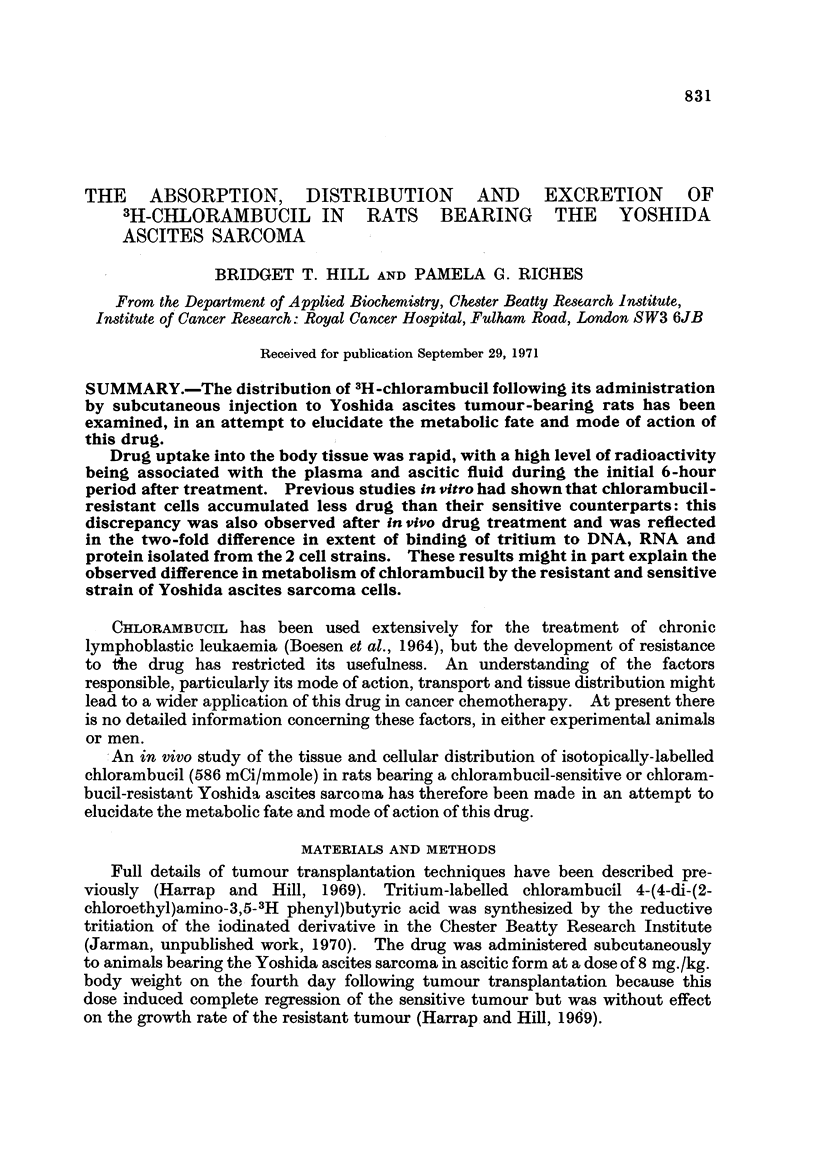

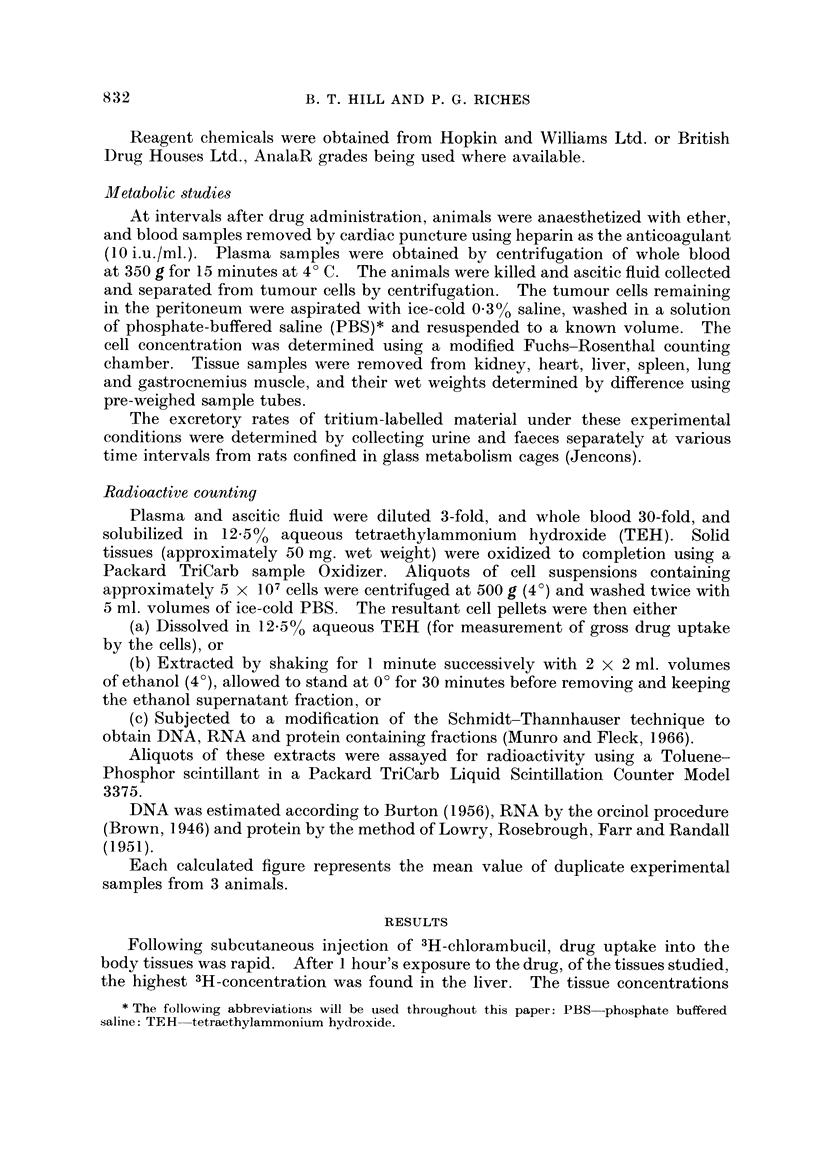

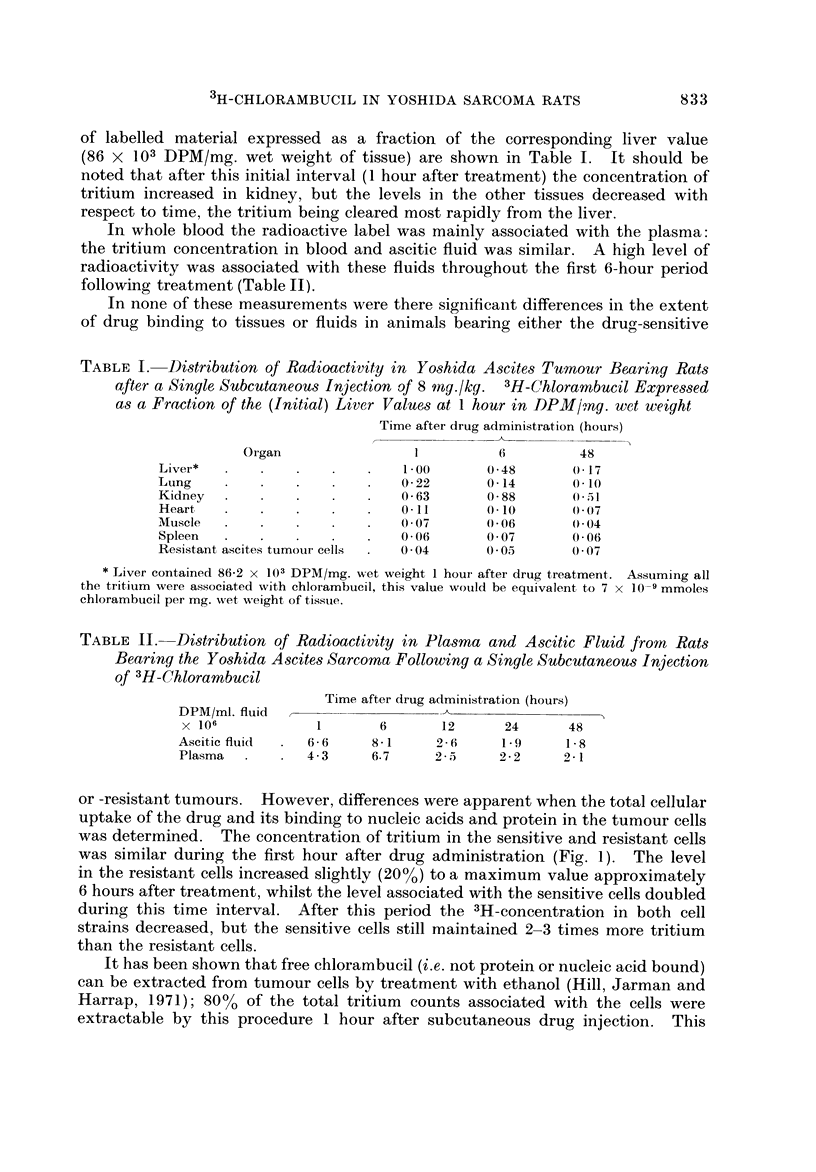

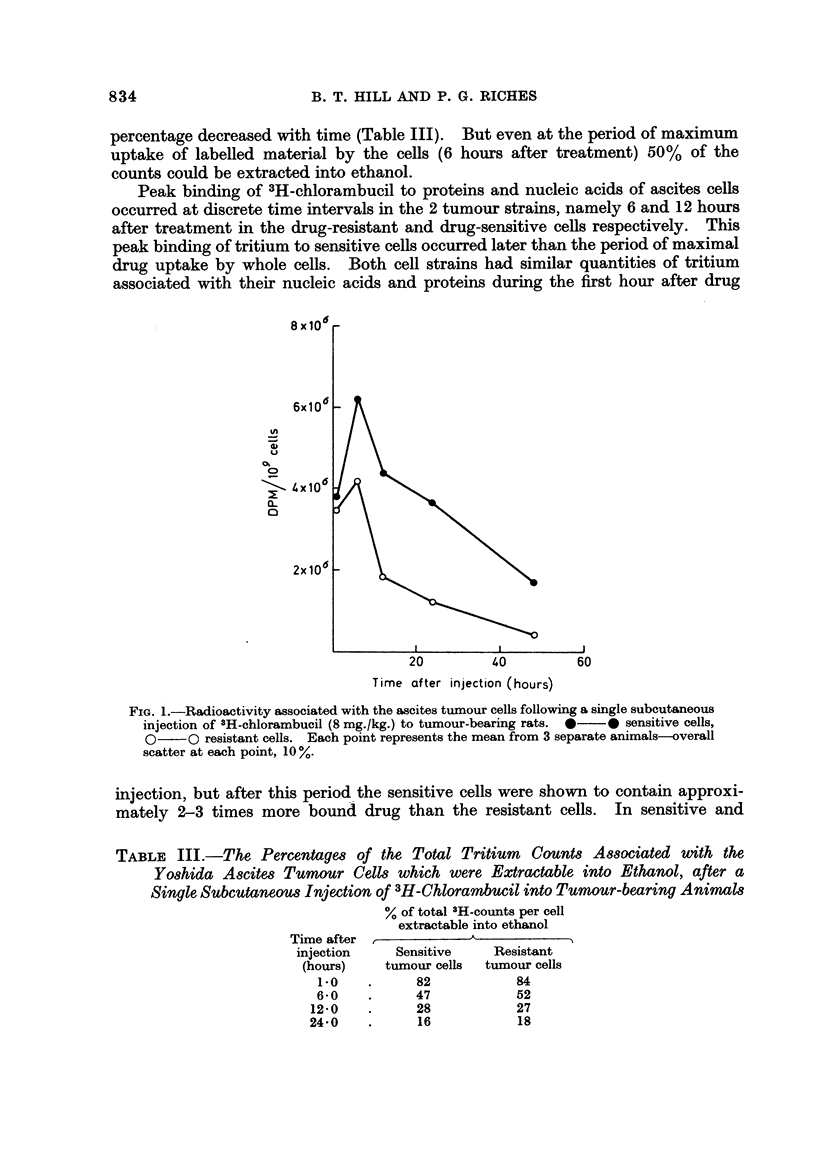

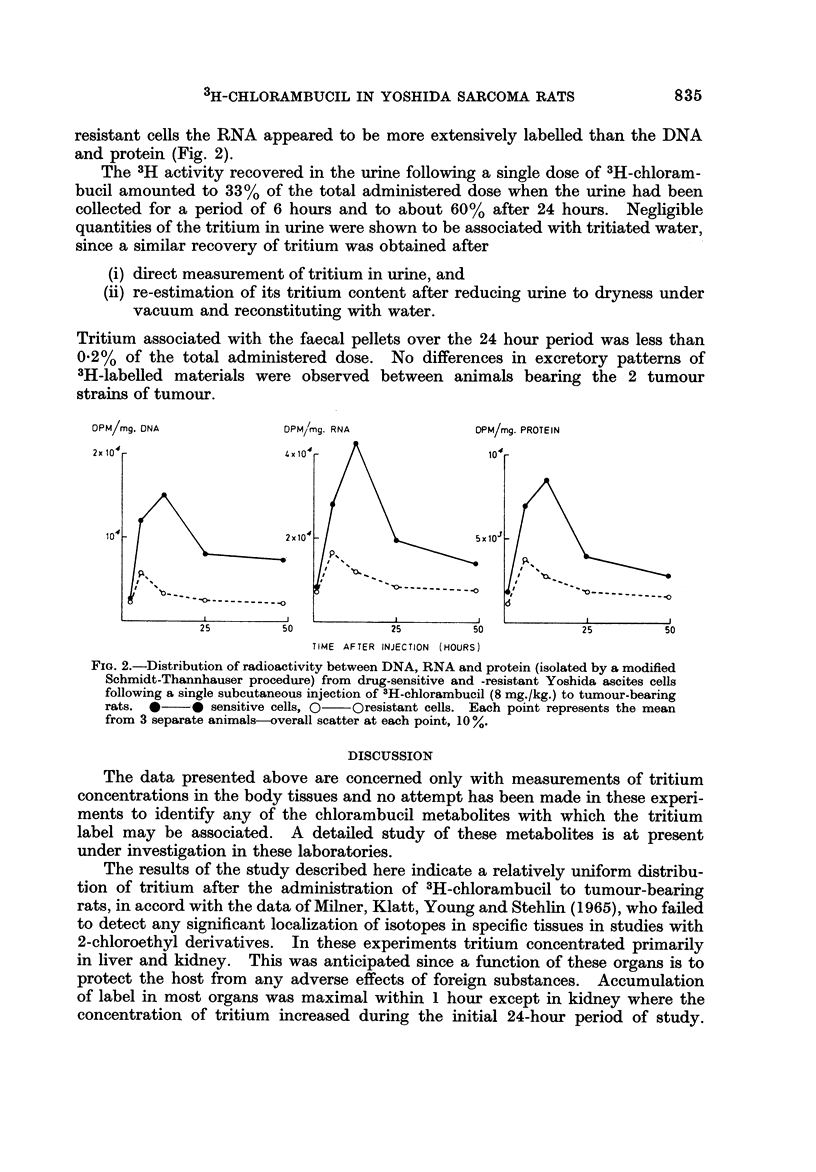

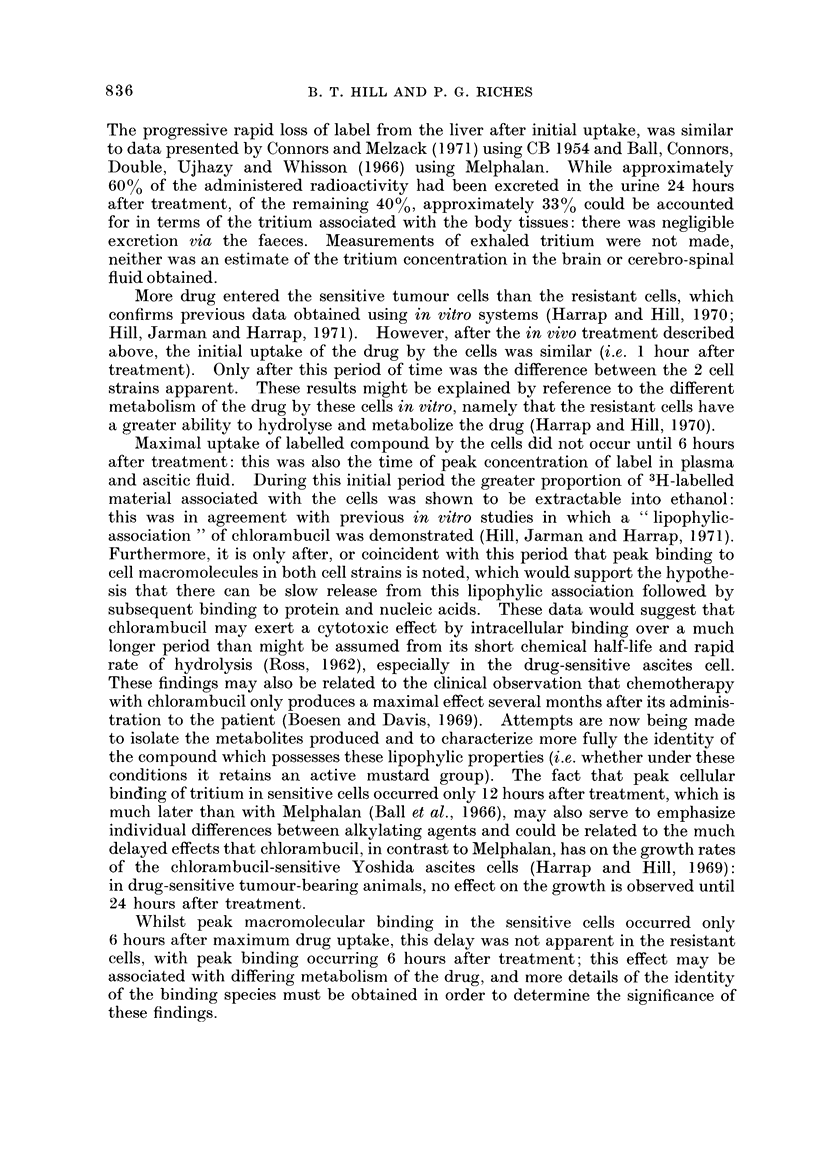

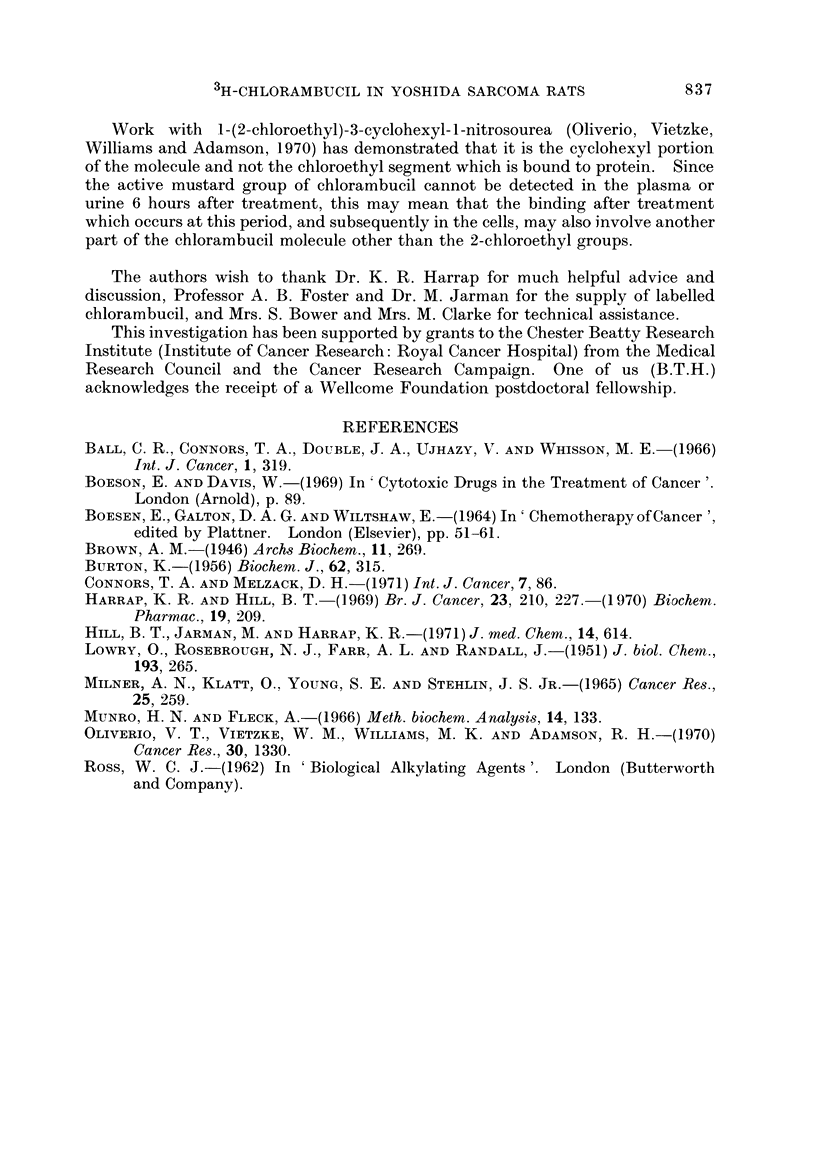


## References

[OCR_00412] BROWN P. S. (1956). Fractionation of human urinary gonadotrophins.. Nature.

[OCR_00400] Ball C. R., Connors T. A., Double J. A., Ujhazy V., Whisson M. E. (1966). Comparison of nitrogen-mustard sensitive and -resistant Yoshida sarcomas.. Int J Cancer.

[OCR_00416] Harrap K. R., Hill B. T. (1969). The selectivity of action of alkylating agents and drug resistance. I. Biochemical changes occurring in sensitive and resistant strains of the Yoshida ascites sarcoma following chemotherapy.. Br J Cancer.

[OCR_00420] Hill B. T., Jarman M., Harrap K. R. (1971). Selectivity of action of alkylating agents and drug resistance. 4. Synthesis of tritium-labeled chlorambucil and a study of its cellular uptake by drug-sensitive and drug-resistant strains of the Yoshida ascites sarcoma in vitro.. J Med Chem.

[OCR_00422] LOWRY O. H., ROSEBROUGH N. J., FARR A. L., RANDALL R. J. (1951). Protein measurement with the Folin phenol reagent.. J Biol Chem.

[OCR_00426] MILNER A. N., KLATT O., YOUNG S. E., STEHLIN J. S. (1965). THE BIOCHEMICAL MECHANISM OF ACTION OF L-PHENYLALANINE MUSTARD. I. DISTRIBUTION OF L-PHENYLALANINE MUSTARD-H3 IN TUMOR-BEARING RATS.. Cancer Res.

[OCR_00432] Oliverio V. T., Vietzke W. M., Williams M. K., Adamson R. H. (1970). The absorption, distribution, excretion, and biotransformation of the carcinostatic 1-(2-chloroethyl)-3-cyclohexyl-1-nitrosourea in animals.. Cancer Res.

